# Fiber Optic Based Distributed Mechanical Vibration Sensing

**DOI:** 10.3390/s21144779

**Published:** 2021-07-13

**Authors:** Vít Novotný, Petr Sysel, Aleš Prokeš, Pavel Hanák, Karel Slavíček, Jiří Přinosil

**Affiliations:** 1 Faculty of Business and Management, Brno University of Technology, 612 00 Brno, Czech Republic; 2 Faculty of Electrical Engineering and Communication, Brno University of Technology, 616 00 Brno, Czech Republic; sysel@feec.vutbr.cz (P.S.); prokes@feec.vutbr.cz (A.P.); hanakp@feec.vutbr.cz (P.H.); slavicekkarel@feec.vutbr.cz (K.S.); prinosil@feec.vutbr.cz (J.P.)

**Keywords:** distributed fiber optic sensor, vibration sensor, mechanical vibrations, ϕ-OTDR

## Abstract

The distributed long-range sensing system, using the standard telecommunication single-mode optical fiber for the distributed sensing of mechanical vibrations, is described. Various events generating vibrations, such as a walking or running person, moving car, train, and many other vibration sources, can be detected, localized, and classified. The sensor is based on phase-sensitive optical time-domain reflectometry (ϕ-OTDR). Related sensing system components were designed and constructed, and the system was tested both in the laboratory and in the real deployment, with an 88 km telecom optical link, and the results are presented in this paper. A two-fiber sensor unit, with a double-sensing range was also designed, and its scheme is described. The unit was constructed and the initial measurement results are presented.

## 1. Introduction

Optical fibers are extensively used in telecommunications, where they can transport data at bit rates in the range of terabits per second. Therefore, optical fibers spread almost across the whole data network infrastructure, i.e., not only in the area of WANs (wide area networks), but they are also implemented in data centers, within the backbone parts of enterprise networks, in complex telecommunication distribution nodes, and also in access data networks (“first-mile”) called FTTx technologies (fiber-to-the x = given point close to the customer, e.g., H = home), mainly in the form of passive optical networks (PONs) [1].

Fiber construction, the principle of its operation (total reflection), and the form of the carrier (light) make the data transmission very safe, reliable, and resistant to many sources of disturbances. Nevertheless, the fiber parameters are partially sensitive to ambient conditions, such as temperature, strain, vibrations, or a strong ambient electromagnetic field, and this has an impact on optical signal propagating through a fiber [2]. These facts predetermine the usage of optical fibers for sensing applications [2]. Many sensor types using optical fibers have been designed and are now used in many areas of industry. In addition, chemical and biochemical sensors are also available. Many biochemical fiber sensors are tapered-based, and they are used to detect labeled target analytes, such as glucose, phospholipase, and many others for human health monitoring [3,4,5,6].

Most sensor applications are point sensors or quasi-distributed, but more and more applications using optical fibers in a fully distributed manner are investigated and implemented. The distributed optical fiber sensors behave as a sensor network that provides spatially resolved distribution of physical quantity measurements along the path of the sensing fiber. Ranges of tens of kilometers or even higher can be reached by various schemes that were designed during the last two decades.

Distributed sensors are used to measure quantities such as temperature, strain, mechanical vibrations, and solutions used to guard pipelines, rails, frontiers, perimeters, and they can have yet more applications [7,8,9,10,11].

The motivation of our research was to design, construct, and verify single- and two-fiber vibration sensors, and to offer them to the industry to verify covering its demands on security and structural health-monitoring solutions. The goal of our effort, and the reason why our project proposal was accepted and funded, was to have our own solution in the Czech Republic, with good performance parameters. The two-fiber sensor variant offers cost-effective usage of expensive optical components, such as a laser, optical high-power amplifier, acousto-optic modulator, etc. We do not know research papers dealing with such a design.

The paper is organized as follows: Section 2 describes optical fiber-based sensor principles and schemes, and Section 3 deals with the system design and experiments, which are then discussed and summarized in Section 4.

## 2. Optical Fiber-Based Sensing

Two groups of optical fiber sensors are defined [2] as follows:

1. Extrinsic (hybrid) fiber optic sensors;
2. Intrinsic fiber optic sensors.

Extrinsic sensors are sensors where the sensing point is located outside the fiber line itself, while the second group of sensors uses the optical fiber itself as a sensory medium.

Optical fiber sensors can be used as follows:

- A point sensor, where there is only one point of sensing or where the quantity is spread equally along with the fiber [2];
- A distributed sensor, where the measured quantity has local influence and the measurement method can localize it so that the fiber trace can represent hundreds or thousands of sensors simultaneously [2].

**Figure 1:**
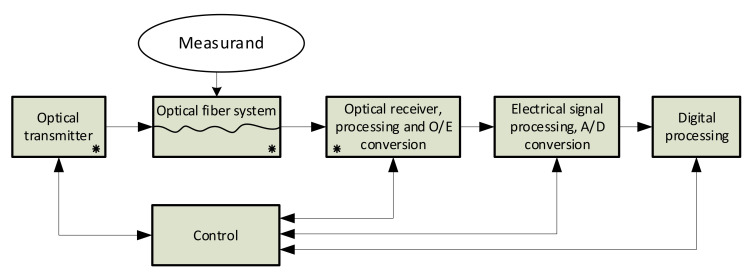
General architecture of modern optical fiber-based sensing system.

A general scheme of an optical fiber-based sensor is shown in Figure 1, as follows:

The “Optical transmitter“ consists of a light source (mainly a laser) and, in the majority applications, also a modulator, amplifiers, filters, and other components to generate a required form of the light signal at an appropriate power level and with a required spectrum. In most cases, the monochromatic and very stable, i.e., a highly coherent, light source is required to obtain high sensor sensitivity and the required accuracy.

The “Optical fiber system” includes one or more fiber segments and the other components, such as optical splitters, couplers, isolators, circulators, and some schemes also contain polarization controllers, polarization splitters, prisms, lenses, and mirrors.

The “Optical receiver, processing, and O/E conversion” block commonly includes optical amplifiers, optical filters, and/or a block of coherent detection that may precede an o/e converter. The o/e converter changes a stream of photons into an electrical signal, i.e., current or voltage. The converter may have a form of one photodetector (a photodiode mostly), or a form of the balanced photodetector (a couple of matched photodiodes connected as a differential circuit) in the case of heterodyne coherent detection schemes.

The “Electrical signal processing and A/D conversion” block further processes the electrical signal and converts it to the digital form. Electrical amplifiers, filters, and, optionally, mixers are included before an A/D conversion.

The “Digital processing” block processes the digital signal form to get the required information that the sensor is designed to provide.

The “Control” block controls the whole measurement process.

### 2.1. Fiber Sensing Principles

Several principles can be used to construct sensors using an optical fiber as the sensory medium. Sensors designed, tested, and used for sensing purposes during the decades, by many scientists and engineers working in this area of research, are documented in [2]. The first group of methods is based on the most straightforward effect, i.e., light intensity change. These sensors are either based on violation of the total reflection principle of light propagation alongside the fiber (pressure or position sensors based on fiber microbending) or (in the case of extrinsic sensors) when the external light propagation parameters change when the light leaves the fiber, reflects from the reflector, and returns back to the detector. A big group of sensors uses a light interferometry principle [12]. They use phase differences between the light beams that travel through the interferometer arms and then meet each other and interfere. The proper function requires interfering beams to be highly coherent, and therefore the difference in the arm lengths must be much lower than the coherence length of the laser source. Fabry-Pérot, Mach-Zehnder, Michelson, and Sagnac are the most known interferometer principles [12]. The interferometric methods are susceptible, but their results do not give information about the location if used as an intrinsic sensor for vibration sensing.

### 2.2. Distributed Optical Fiber Vibration Sensors

**Figure 2:**
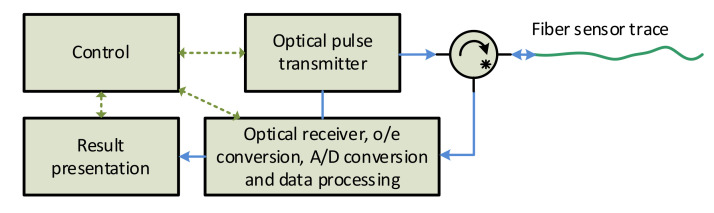
Block structure of the distributed fiber sensor utilizing signal backscattering principle.

Distributed optical fiber vibration sensors have the capability of mechanical vibrations sensing in a distributed manner, i.e., they can detect and localize many events along with the sensing fiber, simultaneously. Many techniques, measurement methods, and schemes were proposed during the last two decades [7,8,9,10,11,12,13,14,15,16,17,18,19,20,21,22,23,24,25,26,27,28,29,30,31,32,33]. The most common measurement scheme is based on the analysis of the Rayleigh backscattered signal from interrogating pulses sent to the fiber, which is similar to the reflectometry principle used in OTDR (optical time-domain reflectometry), as shown in Figure 2.

The standard OTDR technique has been used for decades by the telecom industry, to detect cable breaks, improper fiber splices, or defective connectors [13]. Legacy OTDR is based on a combination of Rayleigh scattering and signal reflections along the fiber irregularities. The core of the OTDR is a wideband light source, mostly the Fabry-Perrot laser source lasing on 1310 nm, 1550 nm, or 1625 nm wavelengths. The typical OTDR output power is approximately 0 dBm and the interrogating pulse width is in the range 0.005–500 μs.

The principle used for vibration sensing is different and is based on the changes in the interference patterns among the discrete scattering centers within half of the optical pulse width [17,21]. The highly coherent laser as the light source is required to reach high sensitivity. The method is called phase-sensitive optical time-domain reflectometry, which is abbreviated as ϕ-OTDR or phi-OTDR [19].

In addition to Rayleigh scattering, other effects can be used for distributed sensing of a temperature or a strain, e.g., Brillouin or Raman ones [16,17].

The sensing fiber may be exposed to mechanical vibrations that change the optical fiber parameters (length, thickness, and refracting index), and so do the parameters of a propagating optical signal. The received signal is processed, converted to the digital form, and a tremendous amount of information can be obtained. The received data is used firstly to detect events, and, in the positive case, the event sources can be localized and classified. The “Time-of-flight” technique is used for localization. Providing the vibration sources act on the fiber in different locations, all of them can be detected and localized simultaneously. Sophisticated signal processing techniques have to be implemented if the event identification is required. The identification methods will be published in a separate paper.

The block scheme in Figure 2 is based on Figure 1. The scheme is based on the original OTDR architecture, except a highly coherent laser is required. The “Optical pulse transmitter” generates narrow optical pulses that are regularly sent to the sensing optical fiber.

**Figure 3:**
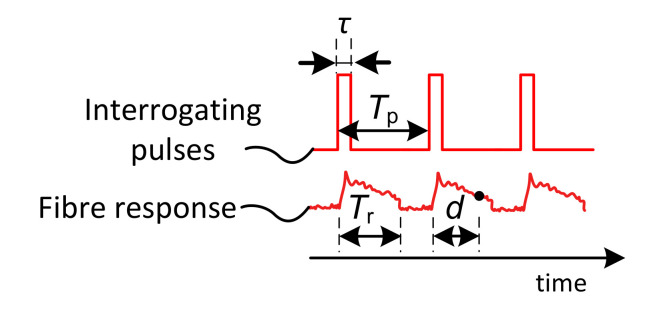
Interrogating optical pulse sequence and the fiber backscattering response.

During the pulse propagation, through the fiber, the optical signal interacts with fiber material inhomogeneities, and the light absorption and scattering effects occur. An elastic scattering effect is called Rayleigh scattering. A portion of this scattered signal is re-captured by the fiber core, and a part of it propagates back to the fiber origin (see Figure 3). The backscattered optical signal is received at the same fiber end, similarly to where the interrogating pulses are emitted in. The instant backscattered signal value is a sum of the responses from particular scattering centers within half of the interrogating pulse width on the fiber. The pulse width therefore determines a sensor spatial resolution. The distributed sensor spatial resolution *R* is given by Formula (1) [14], as follows:

(1) $$R=\frac{\tau v_{g}}{2}$$

where *v*_g_ is the light group velocity in the fiber core (approx. 200,000 km/s for SM silica fiber). Equation (1) clearly shows that the sensor spatial resolution can be improved by narrowing the interrogation pulses. Unfortunately, a narrower pulse at the same power level carries less energy, and therefore shorter sensing ranges are usually the result, because of the limited detector sensitivity and the noise presence. To reach a submeter space resolution, the fast modulators or other switching techniques are required in order to generate nanoseconds or even shorter pulses. Such a requirement may restrict the usage of acousto-optic modulators (AOM), as their rise/fall times are in the tens of ns range, depending on the AOM rf frequency [22,24]. On the other hand, increasing the pulse power to carry more energy may cause nonlinear effects in the fiber and decrease the signal-to-noise ratio.

**Figure 4:**
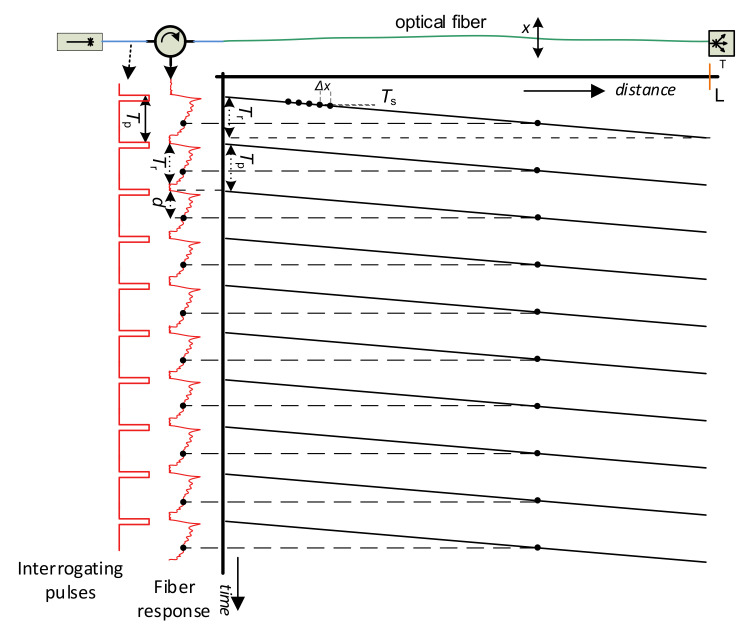
Time–spatial presentation of the fiber sensing and the fiber backscattering response on the sequence of optical interrogation pulses.

Figure 4 depicts a pulse sequence sent to the sensing fiber, and related backscattering responses to them. Provided the light carrier frequency is the same for all the pulses, further restriction arises. The pulse period *T*_p_ has to be longer than the fiber backscattering response *T*_r_; otherwise, the responses from the adjacent pulses would overlap, and the information from the fiber would be partially lost (2) [14].

(2) $$T_{P}\geq \frac{2L}{v_{g}}$$

Thus, for example, when the sensing fiber is 10 km long, the pulse period *T*_p_ must be longer than 100 μs. As a solution for overcoming this restriction, we proposed a solution presented in [34], but we have not implemented it yet.

The latency *d* between the time instances of light pulse transmissions and the instance of the response sample reception (see Figure 4) can be used to calculate the position *x* on the sensing fiber (3) [14], as follows:

(3) $$x\approx \frac{d*v_{g}}{2}$$

Usually, the location *x* on the fiber is impractical for a customer, and hence a geographical location should be obtained. This requires knowledge of sensing cable laying as accurately as possible. Then, a vibration event’s geographical coordinates can be estimated by an interpolation function derived from the precise geographical locations stored in a database.

**Figure 5:**
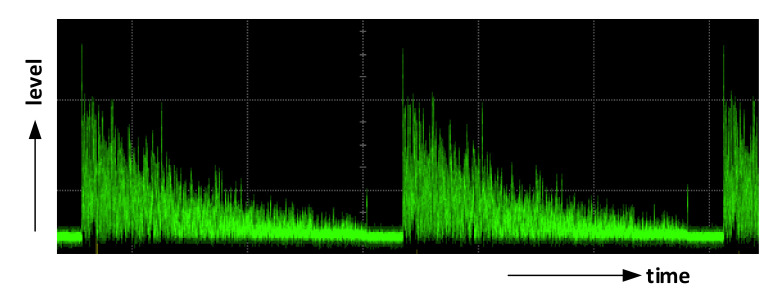
Typical fiber response to narrow spectral linewidth interrogating pulses (result of own experiments).

Figure 5 shows two periods of a standard course of the fiber backscattering response, based on the technique described above before A/D conversion. The signal attenuation and its high fluctuations can be indicated in the figure. The rapid signal attenuation is due to double attenuation process, caused both by the attenuation of the level of interrogating pulses in the forward direction and by the attenuation of backscattered signal level in the backward direction. Sharp backscattered signal fluctuations arise from using a highly coherent laser source, random locations of scattering centers in the sensing fiber, and a random phase of scattered signal contributions generated at scattering centers that interfere within the half pulse width [17]. Due to this effect, and also due to the random signal polarization state of the backscattered signal (in the case of coherent detection, see below), the signal level after the o/e conversion may fall close to the zero value at many time instants, i.e., positions along the fiber, and this deteriorates the sensor sensitivity at corresponding locations along the sensing fiber. A polarization diversity and chirped-pulse phase-sensitive OTDR techniques were proposed to tackle with these issues [14,15].

### 2.3. Distributed Optical Fiber Vibration Sensor Schemes

**Figure 6:**
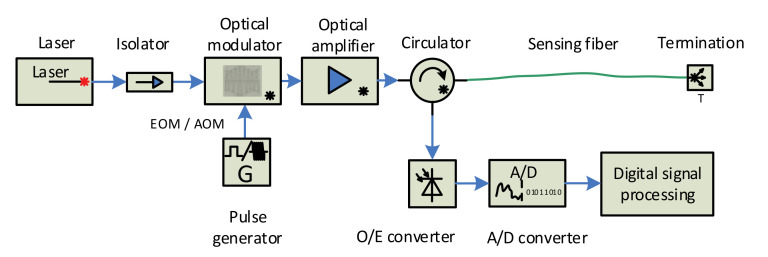
Direct detection scheme.

**Figure 7:**
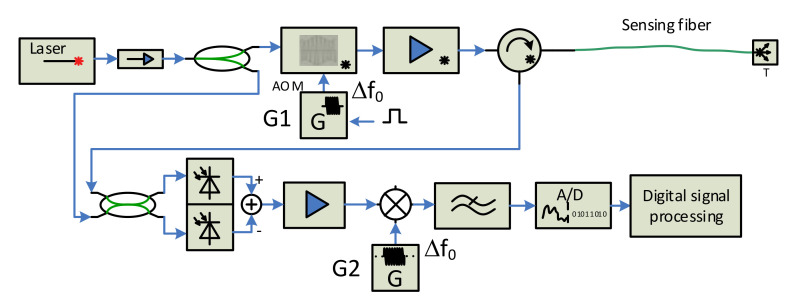
Coherent heterodyne detection scheme.

Two basic schemes are used for backscattered signal reception—direct detection and coherent heterodyne detection, which can be seen in Figure 6 and Figure 7 [29], as follows:

Both schemes contain a laser source that is protected by an optical isolator, followed by an optical amplifier and an optical circulator in the sensor’s forward branch, and an o/e convertor, a data acquisition card, and a computer unit for the digital signal processing in the reception arm. The main difference between the direct detection scheme (Figure 6) and the coherent detection one (Figure 7) consists of applying a local oscillator signal derived from the primary laser, its mixing with the backscattered signal, and applying the balanced o/e converter in which the beat signal amplification process occurs. The heterodyne scheme also contains an analog mixer to obtain a baseband signal if the mixing process is not a part of the digital signal processing.

The direct detection scheme has lower complexity than the coherent one, but it has several issues, described in [21], that limit its performance. As described briefly above, the coherent detection technique consists in the mixing of the backscattered signal with the local optical oscillator and in balanced o/e conversion. The strong local oscillator signal is mixed with a weak backscattered signal, in a 50/50 coupler, and converted to an electrical current in the balanced photodetector. The beat signal generates the current in the balanced photodetector that contains several frequency components. The desired current component is given by Formula (4) [17].

(4) $$I_{1}-I_{2}=2R\sqrt{P_{S}(t)P_{L}}\sin\left[\omega_{\mathrm{AOM}}t+\theta_{S}(t)-\theta_{L}\right]$$

Formula (4) clearly explains the main principle of the weak fiber response amplification. *R* is the convertor responsivity (A/W), *P*_S_ is the power of the received signal, *P*_L_ is the power of the local oscillator, ω_AOM_ is the angular frequency of rf pulses driving the AOM modulator, and *θ*_S_ and *θ*_L_ are the phases of the signal and the local oscillator, respectively. This method improves the sensor sensitivity and provides a higher dynamic range. The cost for these advantages is that an ultra-narrow spectrum light source (laser) with high stability is required. Its spectrum line width has to be in the order of units of kHz or better; thus, the laser is usually the most expensive component of such systems.

**Figure 8:**
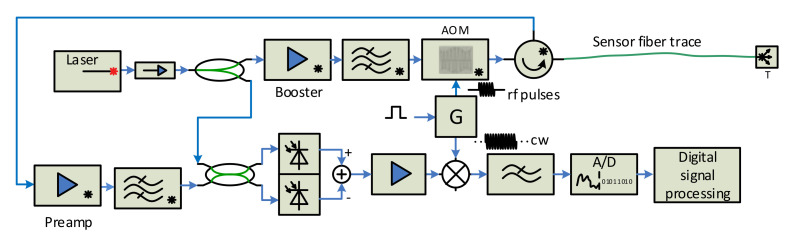
Coherent detection scheme with a single RF signal source.

The sensor performance is influenced by the properties of real components the sensor consists of. Except the laser parameters (ultra-narrow linewidth, frequency and phase stability), the parameters of optical amplifiers, optical modulators and rf generators also have a serious impact on the overall sensor performance. To reach high sensor sensitivity, spatial resolution and sensing length, the sensor design should try to suppress the real component side effects. The addition of an optical bandpass filter behind the optical power amplifier helps by suppressing the ASE noise generated by it. The backscattered signal coming from the sensing fiber is very weak, and adding an optical amplifier to the sensor reception path behind the third port of the circulator, followed by the optical filter (to suppress the ASE noise), will increase the signal power before an o/e conversion. Signal deterioration may also occur if the generators G1 and G2 are not perfectly synchronized and stable. A slight improvement can be reached by using one harmonic generator with a pulse shaper and power amplifier to drive the AOM, as shown in Figure 8. Then, there is only one harmonic generator, and its output is split into two branches. One, continuous wave enters the mixer, while the signal of the second branch is shaped by control pulses, and is appropriately amplified to drive the AOM modulator.

**Figure 9:**
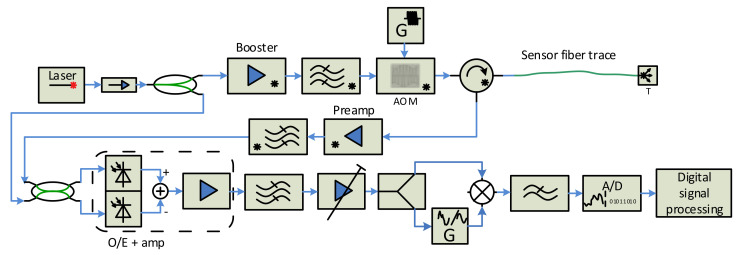
Final sensor unit scheme used of the test-bed.

The rf signal generator driving the AOM is not perfectly stable either. As the fiber response duration is much longer than the width of the radio pulses sent to the AOM modulator, the generator phase and/or frequency may change. The consequence is that the signal transition to the baseband is not perfect and this can cause additional distortion to the signal coming especially from more distant locations of the sensing fiber. In [29], a self-mixing technique was proposed, which uses the amplified backscattered signal for the beat frequency recovery. The scheme implementing this idea is shown in Figure 9. The electrical bandpass filter behind the o/e balanced converter only allows the beat signal to pass, and the following voltage-controlled gain amplifier (VGA) amplifies and equalizes the fiber response characteristics. The VGA output signal is split into two branches then, and one enters the carrier recovery unit before the mixing process. If the beat frequency is correctly recovered, the baseband signal is obtained without a residual frequency shift.

### 2.4. Multi-Fiber Sensor Unit

As the sensor components, such as the laser, the optical amplifiers, the modulator, and the o/e converter, are pretty expensive, it occurred to us that it would be useful to have a scheme, which uses at least some of these elements more efficiently, while adding an acceptable complexity increase. This can be reached by a scheme with more sensing branches, and several reception and processing chains, i.e., the system serving several sensing fibers. The benefit of the multi-fiber solution is a larger area that can be covered by one sensor unit or more complex sensing fiber layouts. The drawback of this design is that more reception chains, more A/D convertors, and a higher computing power are required.

**Figure 10:**
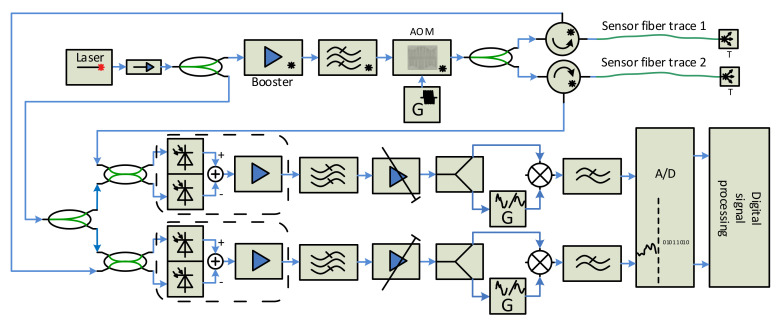
Two-trace sensor unit.

Figure 10 shows a two-trace scheme with two output ports for connecting sensing fibers, and two reception and processing chains, which we designed, constructed and tested, as can be seen in Chapter 0.

The pulse signal generated by the AOM enters a 1 × 2 splitter, and its outputs are connected via two circulators into two sensing arms. The backscattered signals from each fiber are processed separately by the coherent heterodyne detectors and converted to the digital form for digital processing. The solution requires some extra optical components (circulator, couplers, o/e converter), two branches of electrical signal processing, two A/D converters, and a higher computing performance. The benefit is double the sensing length.

## 3. Experimental Setup and Results

Several experimental test-beds were designed and realized to test the principles, as mentioned above. The direct detection architecture was found to be less sensitive than the coherent one, and thus the coherent heterodyne detection system was selected. The resulting design is based on the scheme shown in Figure 7.

**Figure 11:**
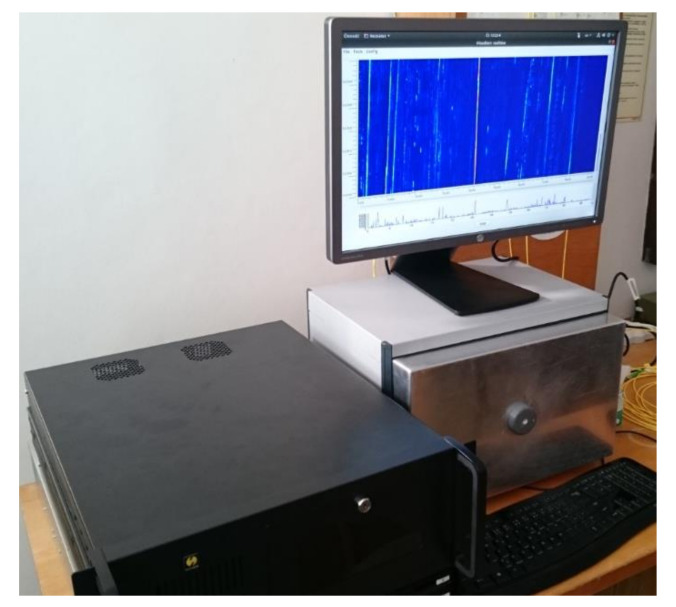
The single-fiber sensor unit testing in a real deployment.

We realized and tested the first version of the interrogation unit in 2015, and the results were presented in [35]. Later, we designed a new version of the sensor probe, according to the scheme from Figure 9, which was constructed in 2018. We used an ultra-narrow laser (10 kHz linewidth) at 1550 nm wavelength, to minimize the signal attenuation (both interrogating pulse and backscattered signal) along the fiber. The pulses are generated by an acousto-optic modulator (AOM), which, in addition to a modulation process with a high extinction ratio (>50 dB [25]), shifts the spectrum of optical signal in the in order of tens MHz [23,24]. We used AOM with a 110 MHz frequency shift. The pulse width can be selected in the range of 100 ns–1 µs. The pulse width corresponds to the spatial resolution (see Formula (1)) in the range of 10–100 m. The generated pulses are amplified by the EDFA booster, filtered by the optical bandpass filter, and sent via the circulator to the sensing fiber. The backscattered optical signal from the fiber returns via the optical circulator and enters the EDFA preamplifier, and, after filtering, it is mixed with a local optical oscillator (OLO) signal, derived from the laser output in the 50/50 coupler. The resulting optical signal is converted to the electrical form, by the balanced o/e converter containing a couple of matched PIN photodiodes and the transimpedance amplifier. The output electrical signal is amplified again and equalized by the logarithmic VGA (variable gain amplifier), and then split into two branches. The signal of one is used to recover the beat carrier, i.e., 110 MHz carrier, which is then mixed with the second branch signal. High-frequency components (above 10 MHz) of the mixer output signal are filtered out, and the resulting signal is converted to the digital form by the A/D converter, with a sampling frequency of 50 MSPS. The obtained data are processed by additional filtering and averaging techniques. The system was constructed and tested both in the laboratory (fiber coils of 20 and 50 km lengths were used as the sensing fibers) and in the field, where the sensor unit was connected to the real optical telecom links, see Figure 11.

**Figure 12:**
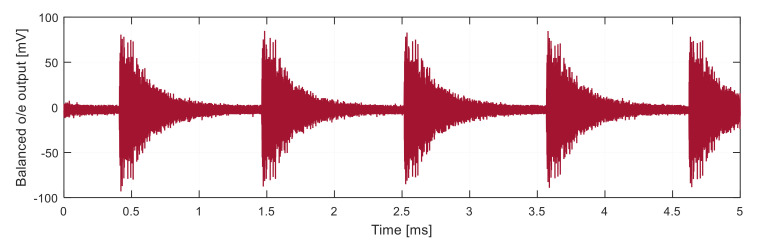
The beat signal at the output of balanced o/e converter.

The beat signal after the o/e conversion is shown in Figure 12.

**Figure 13:**
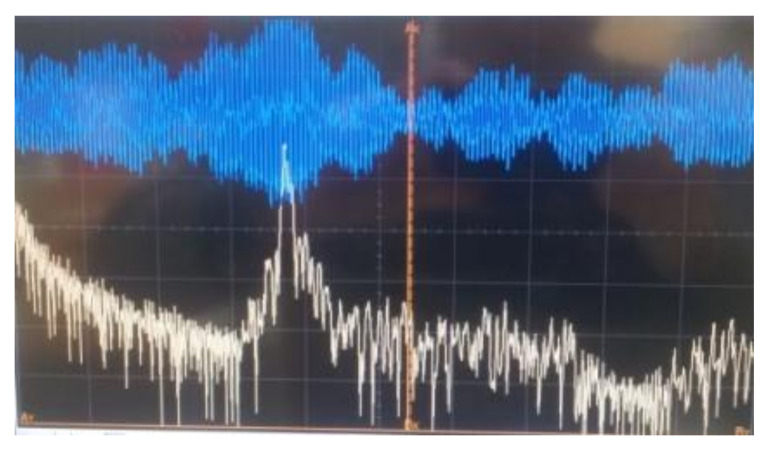
Signal detail after an o/e conversion and its spectrum—coherent heterodyne detection system.

A small segment of the beat signal from the o/e converter is shown in Figure 13. The required signal is its envelope, which is obtained after mixing with the beat frequency and lowpass filtering, as described above. We recovered the carrier from the received beat signal, as depicted in Figure 9.

**Figure 14:**
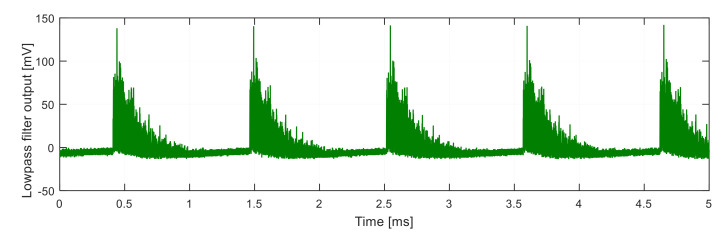
Signal after demodulation without equalization.

After low-pass filtering, the signal shown in Figure 14 is obtained.

**Figure 15:**
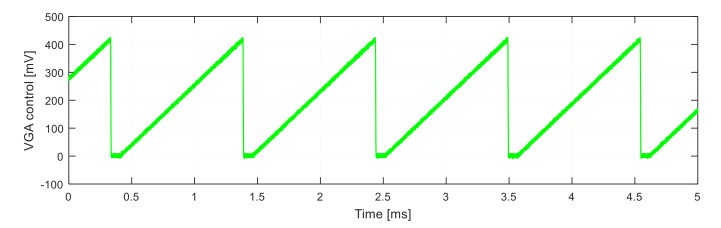
VGA control voltage.

Due to the exponential attenuation of the fiber response, the signal level is relatively weak from large distances. Therefore, the fiber response equalization was proposed and implemented. To equalize the response, the beat signal was amplified by the logarithmic variable gain amplifier (VGA), whose gain was controlled by the sawtooth-shaped signal synchronized with the pulse generation (see Figure 15).

**Figure 16:**
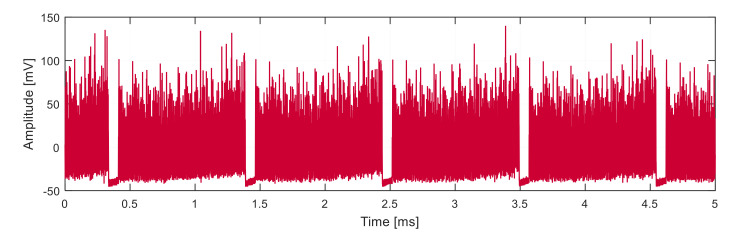
Signal after demodulation and equalization.

After equalization and demodulation processes, and lowpass filtering (see Figure 16), the signal is converted to the digital form for digital processing.

**Figure 17:**
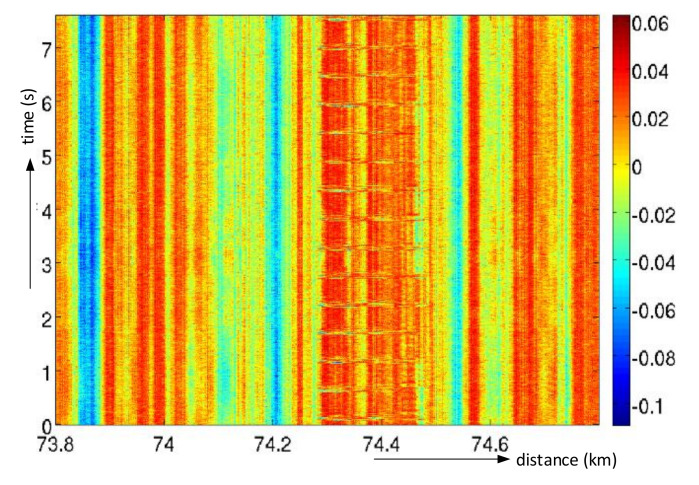
A “waterfall” of raw captured data.

To display the situation along the sensing fiber trace within a period, the software was designed to arrange the fiber responses in a colored two-dimensional matrix, where the color represents the signal levels. Such a signal representation is called “waterfall” in the raw form, and this is obtained. Two-dimensional signal displays information along with the fiber (horizontal axis) and in time (vertical axis, the latest response is at the top), as shown in Figure 17 (only a narrow part of the waterfall in the horizontal direction with a static event occurrence within 7 s is shown). For example, a hammer stroke on a rail was detected at the distance of 74.4 km alongside the fiber.

**Figure 18:**
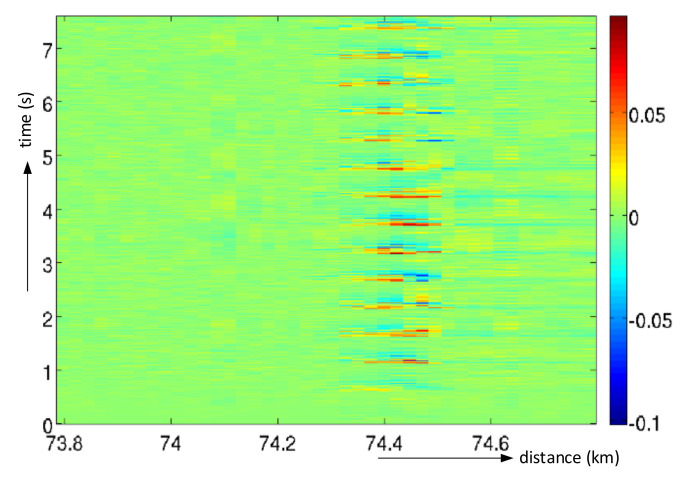
An example of a “waterfall” with a detected event after filtering.

Using FIR filtering with linear phase response along the time axis, the vibration events along the fiber can be emphasized, while the quiet areas are cleaned, as shown in Figure 18.

**Figure 19:**
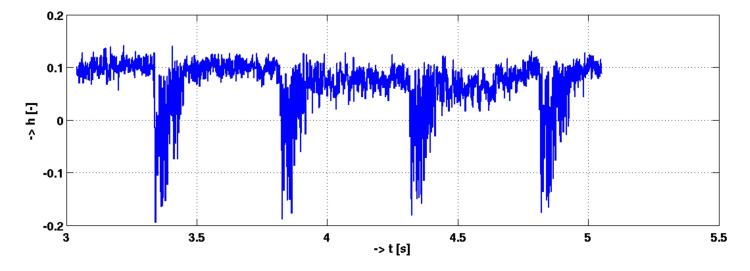
Time-domain signal amplitude evolution from the event location—hammer strokes.

The corresponding time-domain course of the signal (hammer strokes) from the single point in the fiber is shown in Figure 19.

The sensing optical fiber of the field deployment was a dark fiber of a standard telecommunication optic cable trace, of a total length of 88 km, laid along the railway line.

**Figure 20:**
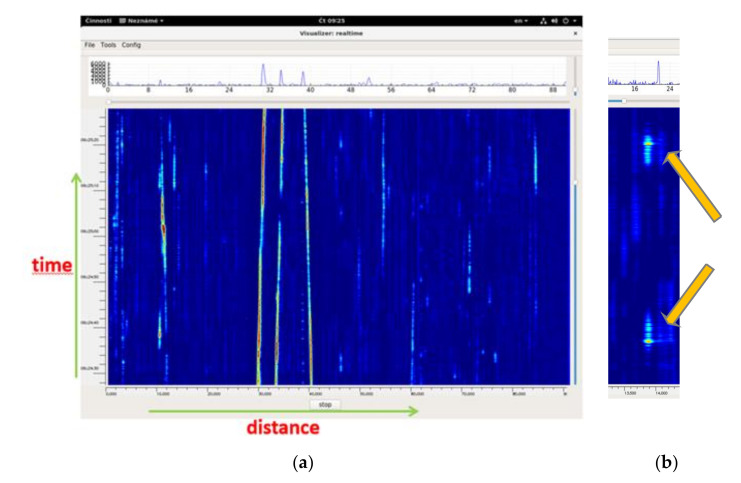
Waterfall with the occurrence of (**a**) multiple simultaneous events—“waterfall”, (**b**) two events when a vehicle is passing a rail crossing.

The waterfall with events moving along the sensing fiber (88 km long) is depicted in Figure 20a. Due to the distributed sensing principle, several simultaneous events can be detected and separately processed. Figure 20b shows two cases of a vehicle passing a rail crossing, each with a different direction of passing.

**Figure 21:**
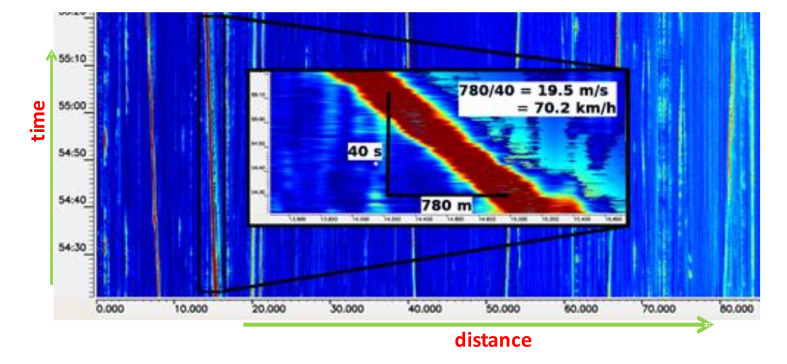
The waterfall with moving vibration sources (trains) and the source movement speed estimation.

The speed of vibration source movement can be derived from the slope of the track in the waterfall, as shown in Figure 21. However, there can be trouble in establishing the speed precisely. First, we need to determine the reference points on the fiber, at particular time instants. The following problem may occur in both cases: when the sources produce too strong or too weak vibrations, compare Figure 21 and Figure 20a. In the first case, the track is broad, and its width changes, and in the second case, the track vanishes at some locations, so that the extrapolation method is required. The sensing fiber along the railways or roads bends and rolls, so it can cause the calculated distance segments within the time periods to differ significantly. After the locations at different times, instants along the fiber are obtained from the detection process. Then, the geographical coordinates have to be determined. That is why the precise geolocation information about the sensing cable laying is required.

**Figure 22:**
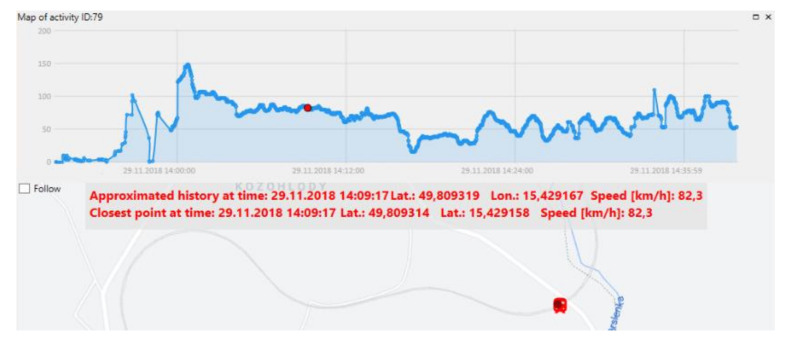
The speed profile of the train and its map location at selected time instant.

The train speed profile can be obtained when the sensing cable is laid down along the railway line, as shown in Figure 22. Thus, the system can provide helpful extra information if required.

**Figure 23:**
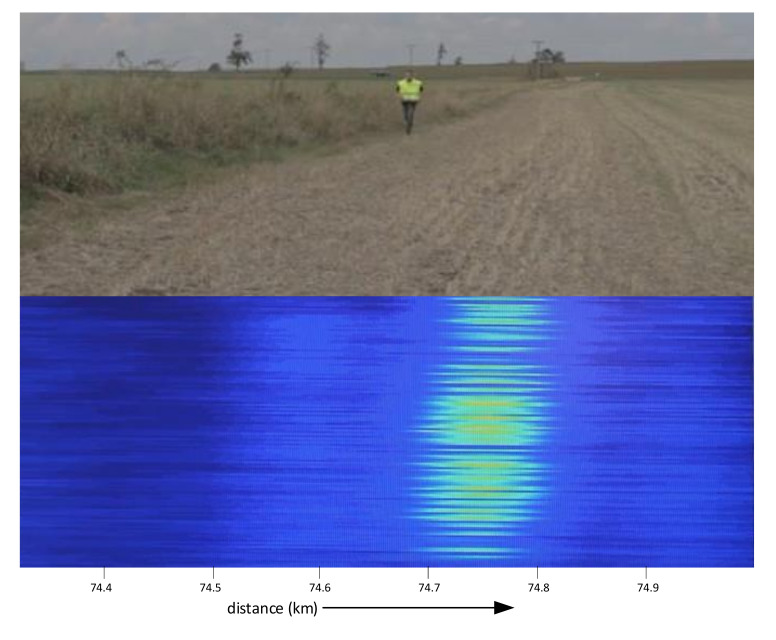
Detection of running person at the distance 74.75 km.

Not only strong vibration sources, but also a human or bigger animal presence in the vicinity of the sensing fiber, is reported by the sensing system, as shown in Figure 23. The optical pulse power was 23 dBm, and the pulse width was 1 µs. The sensing cable was a standard SM telecom cable, laid approximately 1 m under the ground surface. Therefore, it can be seen that the signal influenced by the running person is reliably detectable, including the direction of movement.

### Two-Fiber Sensor Unit Design

**Figure 24:**
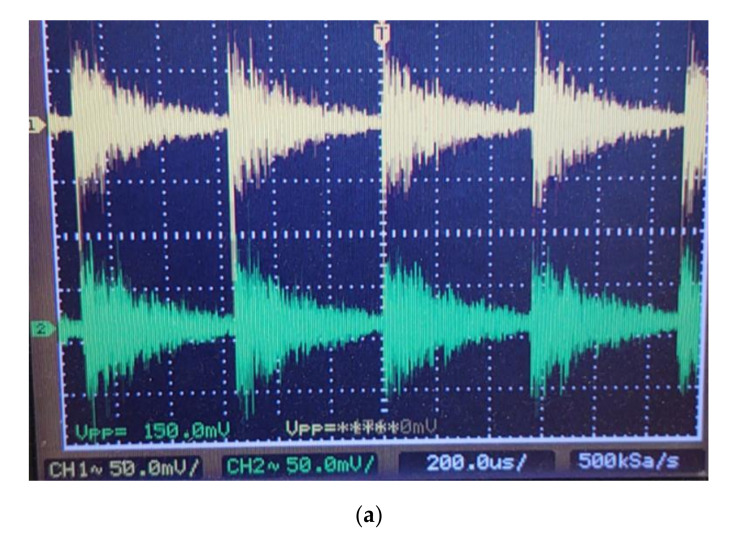

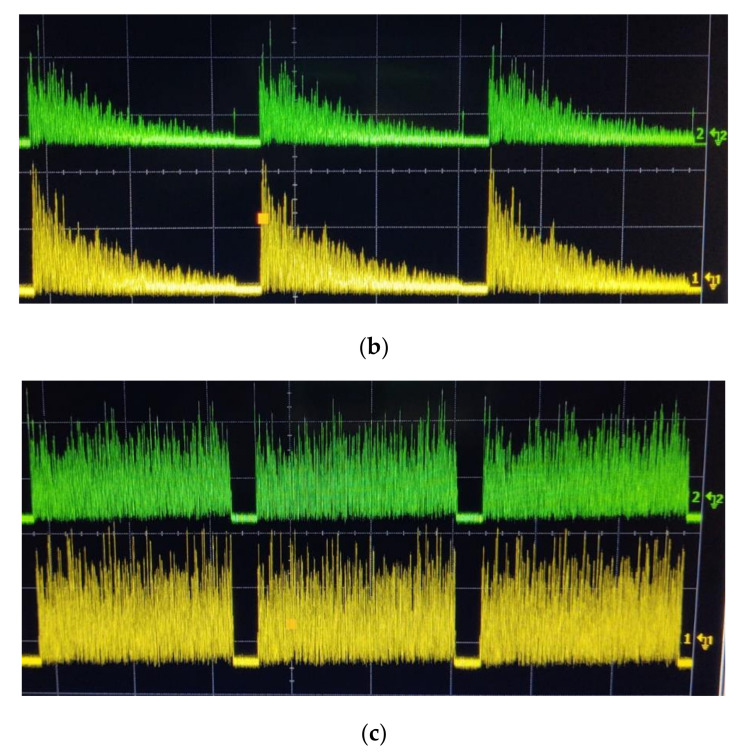
Two-fiber sensor signals obtained from two 50 km links: (**a**) signals at the outputs of o/e convertors, (**b**) signals after mixing and low-pass filtering without equalization, (**c**) equalized traces ready for A/D conversion.

The two-fiber sensor unit, designed according to the scheme depicted in Figure 10, was also constructed and tested. The system testing was accomplished in the laboratory. The system is working correctly, as shown in Figure 24. Two fiber coils, 50 km each, were used as sensing traces. Both pictures, Figure 24a,b, show that the ends of both fibers are detectable, even without an equalization. A final form of analog signals before the A/D conversions is shown in Figure 24c.

**Figure 25:**
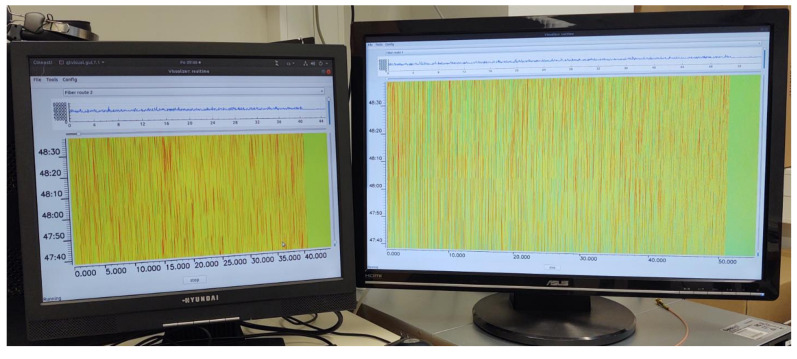
Raw waterfalls from the two sensing fibers—40 and 50 km long.

Raw waterfalls (before other digital processing) from the sensing fibers that are 40 and 50 km long, depicting a simultaneous two-fiber operation, can be seen in Figure 25. It is evident, from the vertical time axis scale, that the system is in a stable state for more than 10 s intervals, even if the sensing fiber is a coiled bare fiber with 250 µm primary coating only. We suppose that the signal will be even more stable when the sensing fiber is in the cable that is buried in the soil.

**Figure 26:**
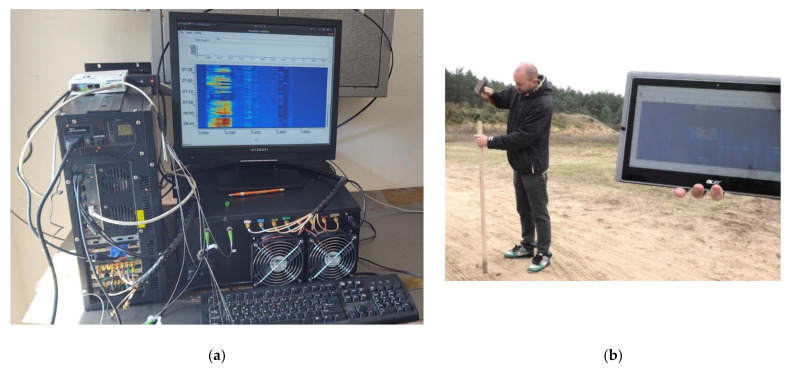
Two-fiber system testing under real conditions: (**a**) the sensing unit; (**b**) the generation of vibrations and the measurement remote control.

The ready sensor unit consisting of interrogation and computer modules, and shown in Figure 26, was tested a few weeks ago in real deployment. The test sensing trace contains a Mini LT Flat Drop 09/125 cable that is directly buried underground. Two fibers of the cable were connected to the system simultaneously. The system was working according to our expectations, but we could not verify its maximum sensing length (we suppose that it can be more than 150 km with two sensing fibers) because of the short sensing traces available. To allow us to control the sensor unit remotely, we designed a software application that offers the user the interface, through which the user in the field with a tablet or a notebook connected to the internet can simultaneously generate the vibrations and watch the system response (Figure 26b).

A data connectivity of several Mbps is needed to control the measurements and to watch the waterfall fluently. A number of different events (rod hammering, ground digging, person walk and run, car movement, etc.) were generated, and many data were collected and used to feed the event classifier machine learning. As this process is quite complex, a lot of samples (hundreds or even thousands) of each event had to be collected. Some details about the event identification process and about the classification model learning will be presented in a separate paper.

## 4. Discussion and Conclusions

The distributed vibration sensing system, based on Rayleigh backscattering and the coherent detection principle, was designed and constructed. It is capable of detecting and localizing many vibration sources along the sensing fiber simultaneously, both static and moving ones. The maximum sensing length reachable by our single-fiber system is above 90 km, and more than 150 km with the two-fiber sensor unit. Thanks to the classification software development and its implementation into our system, several types of vibration sources can be identified—the movement of trains, cars, persons (jumping, walking, running), ground digging, drilling, hammering. The single- and two-fiber sensor units described in the paper are the parts of a more complex sensing system, which we designed, and we are working on putting the whole system into operation. The system testing started at the end of last year and its results are described in [36]. The system is scalable, i.e., it can run more sensing units concurrently, and the information from the sensors can be provided to several independent customers. The information from the sensing process is available through a secure connection of a customer to the system gateway.

Our work has proven the maturity of the used technology. Utilization of Rayleigh backscattering, stimulated by an acoustic wave interfering with the fiber optics cable, is a viable approach for vibration sensing and linear structures surveillance. Even though the technology is still pretty expensive, it has unique capabilities, especially the long reach. Therefore, the sensing system based on this technology seems very promising for the surveillance of large linear structures, such as oil and gas pipes, railways, and similar, as proven by our field measurement.

Our further research will be oriented on the optimization of the system performance. The main tasks are investigating the necessary laser source performance, the optimization of interrogating pulse generation, a more efficient weak fiber response amplification process, further improvement of signal filtering, and more sophisticated digital signal processing and event classification methods.

## Data Availability

No public data resources were used, all data used in the research was obtained from own measurements.
